# Composition of Triterpenoids in *Inonotus obliquus* and Their Anti-Proliferative Activity on Cancer Cell Lines

**DOI:** 10.3390/molecules25184066

**Published:** 2020-09-06

**Authors:** Jaecheol Kim, Si Chang Yang, Ah Young Hwang, Hyunnho Cho, Keum Taek Hwang

**Affiliations:** 1 Department of Food and Nutrition, and Research Institute of Human Ecology, Seoul National University, Seoul 08826, Korea; ddeol@snu.ac.kr (J.K.); sich0502@snu.ac.kr (S.C.Y.); gkhd874@snu.ac.kr (A.Y.H.); 2 Food Analysis Team, Busan Metropolitan City Institute of Health and Environment, Busan 46616, Korea; atrac3p@snu.ac.kr

**Keywords:** *Inonotus obliquus*, chaga mushroom, triterpenoids, cancer cells, anti-proliferative activity

## Abstract

The objective of this study was to determine the composition of triterpenoids in the extracts from the inner and outer parts of *Inonotus obliquus* and to evaluate their anti-proliferative activity against cancer cell lines (HT-29, AGS, MCF-7, and PC3). Inner and outer parts of *I. obliquus* were extracted with 80% methanol for 24 h. The extract was fractionated by Diaion HP-20 resin to obtain the triterpenoid fraction. Composition of triterpenoids in the fraction was analyzed by HPLC and LC-ESI-MS. Anti-proliferative activity was evaluated by MTT assay against cancer cell lines. Inotodiol and trametenolic acid were major triterpenoids in both of the inner and outer parts of *I. obliquus*. Inotodiol in triterpenoid fractions from the inner and outer parts of *I. obliquus* was 153.9 ± 15.4 mg/g (dry basis (db)) and 194.1 ± 11.5 mg/g, respectively. Trametenolic acid in triterpenoid fractions from the inner and outer parts of *I. obliquus* was 94.5 ± 9.15 mg/g (db) and 106.3 ± 8.23 mg/g, respectively. Triterpenoids in the outer part were significantly higher than those in the inner part. Anti-proliferative activity of the triterpenoid fraction from the outer part against AGS, MCF-7, and PC3 was also significantly higher than that of the inner part.

## 1. Introduction

*Inonotus obliquus*, called chaga in Russia and kabanoanatake in Japan, is a white rot fungus that belongs to the family of Hymenochaetaceae [1]. It has been used as a traditional medicine for cardiovascular diseases, diabetes, and gastro-intestinal cancers without toxicity in Russia, Poland, and Baltic countries since the 16th century [2]. Although clinical trials with *I. obliquus* have been scarce, the safety and efficacy of the mushroom may be evidenced with its traditional use for centuries [3]. Recently, the extracts from this mushroom have been known to have biological activities such as anti-cancer, anti-inflammatory, and anti-oxidant activities [4,5,6]. Previous studies have reported that triterpenoids in *I. obliquus* have a significant anti-cancer effect on various cancer cells [7,8]. Ma et al. (2013) also reported that the petroleum ether fraction from *I. obliquus* containing triterpenoids was more active against cancer cells than other solvent extracts of *I. obliquus* [9]. However, most of these studies have focused on crude extracts or organic solvent fractions of *I. obliquus*. It has hardly been concluded that the anti-cancer activity is directly related to triterpenoids in *I. obliquus* because phenolic compounds, polysaccharides, and proteins are also present in crude extracts. Therefore, the fractionation process may be needed to demonstrate the anti-cancer effect of triterpenoids in *I. obliquus*. Solid-phase extraction can be a more appropriate method than solvent fractionation to obtain the triterpenoid fraction in extracts. Oleszek et al. (2002) used a C18 micro-column to obtain triterpenoid saponins from *Trifolium* seeds [10]. Li et al. (2007) also used a Diaion HP-20 column to remove phenolic compounds and obtain a triterpenoid fraction from *Acanthopanax senticosus* [11].

The outer part of *I. obliquus*, which has an appearance similar to rough and crispy black charcoal, is usually discarded before extraction or pulverization in the industry because it has been reported to have no biological activities [12]. Nakajima et al. (2007) also reported that the anti-oxidant activity of *I. obliquus* was higher in the extract of the fruiting body (inner part) than that of the sclerotium (outer part) [13]. Zhong et al. (2009), however, reported that triterpenoids which have anti-virus activity exist mainly in the outer surface of *I. obliquus* [14]. The composition and biological activities of triterpenoids in the outer part of *I. obliquus* have been little studied.

The purpose of this study was to identify triterpenoids in the inner and outer parts of *I. obliquus*. In addition, anti-proliferative activities of methanol extracts and triterpenoid fractions from the inner and outer parts of *I. obliquus* (IPTF and OPTF, respectively) were comparatively determined.

## 2. Results and Discussion

### 2.1. Extraction Yields of IPTF and OPTF

Extraction yields of the IPTF and OPTF were 0.51 ± 0.04% and 0.78 ± 0.14% on a dry basis, respectively (Table 1). The extraction yield of the OPTF in this study was quite similar to that of an ethyl acetate extract (0.72 ± 0.03%, dry basis) in a previous study [6]. The previous study also reported that the extraction yield of bark from *I. obliquus* was higher than that of its inside.

### 2.2. Total Triterpenoids in I. obliquus

Total triterpenoids in the OPTF (554.5 ± 13.9 mg ursolic acid equivalent (UAE)/g extract) were significantly higher (*p* < 0.05) than those in the IPTF (469.2 ± 10.2 mg UAE/g extract), even when calculated on the basis of the raw materials (Table 1). Wang et al. (2014) also reported that total triterpenoids in methanol extracts of birch core and bark of *I. obliquus* were 2.36 and 2.43 mg/g raw material, respectively [6].

However, the method for determining total triterpenoids in the present study might not exclusively detect triterpenoids only because the reagents (perchloric acid and vanillin) used in the method can react with other compounds which have unsaturated bonds [15]. To more accurately determine triterpenoids, LC-MS was performed.

### 2.3. Composition of Triterpenoids in I. obliquus

**Figure 1:**
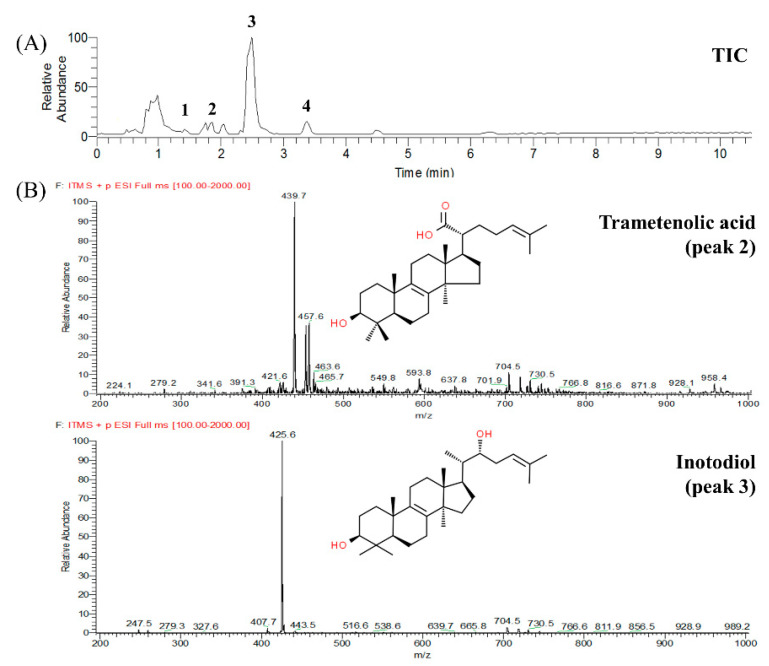
LC-ESI-MS total ion chromatogram (TIC) (**A**) of triterpenoid fraction from *Inonotus obliquus* and mass spectra (**B**) of peaks 2 (trametenolic acid) and 3 (inotodiol) shown in the TIC.

**Figure 2:**
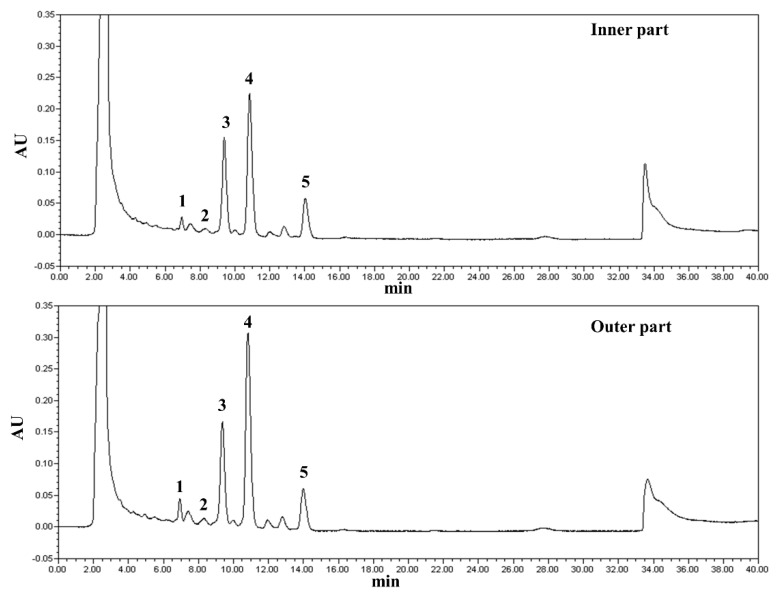
HPLC chromatograms of triterpenoid fractions from inner and outer parts of *Inonotus obliquus*. Peak 1, betulinic acid; 2, betulin; 3, trametenolic acid; 4, inotodiol; and 5, unknown.

The total ion chromatogram and MS spectra of the IPTF and OPTF are shown in Figure 1. Triterpenoids were identified from their protonated molecular ions [M + H]^+^. Four peaks were observed to be major components in the IPTF and OPTF. Mass values of peaks 1 and 2 in Figure 1 were 457 [M + H]^+^ and 439 [M + H − H_2_O]^+^, respectively. These mass values were also the same as those of betulinic and trametenolic acids [16]. Retention time (RT) of peak 1 on the HPLC was the same as that of the betulinic acid standard (Figure 2), suggesting peak 1 be betulinic acid. Peak 2 was determined as trametenolic acid referring to the previous study [16]. The elution order of betulinic and trametenolic acids on the HPLC was similar to the result of a previous study [17]. Peak 3 might be lupenone or inotodiol considering its mass value which was detected at *m*/*z* 425. As the molecular weights of lupenone and inotodiol are 424 and 442, respectively, they could have *m*/*z* 425 ([M + H]^+^ and [M + H − H_2_O]^+^ ion fragments, respectively). To exactly confirm peak 3, lupenone and inotodiol standards were analyzed by the HPLC. Peak 3 was identified as inotodiol because its RT and UV spectrum on the HPLC corresponded to those of the inotodiol standard. Peak 4 could not be identified.

HPLC chromatograms of triterpenoids in the IPTF and OPTF are shown in Figure 2. RTs of the peaks on the HPLC chromatograms (Figure 2) were not the same as those of the peaks on the LC-MS chromatograms (Figure 1) because the instruments and columns used for the HPLC and LC-MS analyses were different. Peaks 1, 3, 4, and 5 detected by the HPLC (Figure 2) were betulinic acid, trametenolic acid, inotodiol, and an unknown compound, respectively. Peak 2 was determined as betulin by matching the RT and UV spectrum of the peak with the betulin standard. The peaks were quantified using betulin, betulinic acid, and inotodiol as standards (Table 2). Contents of peaks 3 and 5 were calculated as betulinic acid equivalent. There was no big difference in triterpenoid constituents between the IPTF and OPTF. However, the content of triterpenoids in the OPTF was significantly higher than in the IPTF (*p* < 0.05). This might be because contents of betulinic acid and inotodiol were significantly higher in the OPTF (*p* < 0.05). Major triterpenoids in *I. obliquus* were trametenolic acid, inotodiol, and an unknown compound. The three compounds in the IPTF and OPTF accounted for 94.8 and 94.0%, respectively, of the total triterpenoids quantified by the HPLC. This result is similar to a previous report [17], in which inotodiol and trametenolic acid were major components in *I. obliquus*. Du et al. (2011) also reported that contents of inotodiol and trametenolic acid in *I. obliquus* were 130 and 70 mg/g extract, respectively [18].

### 2.4. Anti-Proliferative Activity of Triterpenoid Fractions from I. obliquus

In this study, concentrations up to 300 µg/mL of the triterpenoid fractions did not significantly affect the normal cells (RAW 264.7 cells) (data not shown). Therefore, the MTT assay of triterpenoid fractions against cancer cell lines was performed at a concentration of 300 µg/mL or less.

**Figure 3:**
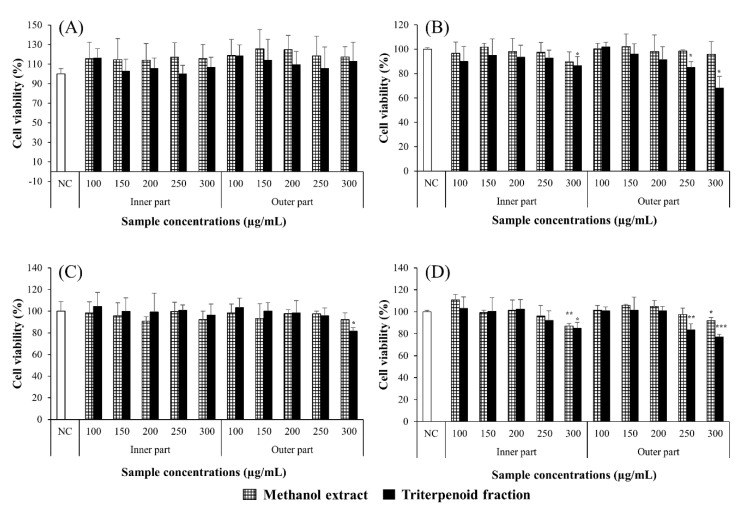
Anti-proliferative activities of triterpenoid fractions and methanol extracts of *Inonotus obliquus* on HT-29 (**A**), AGS (**B**), MCF-7 (**C**), and PC3 (**D**) cancer cell lines. Bars represent means and standard deviations of three independent experiments. * *p* < 0.05, ** *p* < 0.01, and *** *p* < 0.001 compared to negative control (NC) treated with non-serum medium containing 0.1% DMSO.

Anti-proliferative activities of the methanol extracts and the triterpenoid fractions were determined against various cancer cell lines (Figure 3). All the extracts and fractions were not able to significantly inhibit proliferation of HT-29 cells. The triterpenoid fractions had dose-dependent anti-proliferative activity on AGS, MCF-7, and PC3 cells. The highest concentration of the triterpenoid fractions and even 250 µg/mL of the OPTF significantly inhibited proliferation of AGS cells. Proliferation of MCF-7 cells was significantly inhibited at 300 µg/mL of the OPTF (*p* < 0.05). The highest concentration of the methanol extract and triterpenoid fraction of the inner part of *I. obliquus* significantly inhibited proliferation of PC3 cells (*p* < 0.01 and *p* < 0.05, respectively). The OPTF was able to significantly inhibit proliferation of PC3 cells at 250 and 300 µg/mL (*p* < 0.01 and *p* < 0.001, respectively). However, only the highest concentration of the methanol extracts from the inner and outer parts of *I. obliquus* significantly inhibited proliferation of PC3 cells (*p* < 0.01 and *p* < 0.05, respectively). Thus, the triterpenoid fractions might have higher anti-proliferative activity on cancer cells than the methanol extracts and the effects of the OPTF were better than those of the IPTF. This is in accordance with the result of Ma et al. (2013) [9], reporting that a petroleum ether extract of *I. obliquus*, which had a large amount of triterpenoids, was the most active against cancer cell lines among butanol, ethanol, ethyl acetate, petroleum ether, and water extracts. Moon and Lee (2009) also reported that extracts containing a higher amount of hydrophobic substances had a stronger anti-cancer activity on various cancer cell lines [19]. Cha et al. (2007) reported that viability of AGS and MCF-7 cells fell below 80% when treated with a water extract of *I. obliquus* at a concentration of 1 mg/mL [20].

## 3. Materials and Methods

### 3.1. Chemicals and Reagents

Betulin, betulinic acid, ursolic acid, and vanillin were purchased from Tokyo Chemical Industry Co., Ltd. (Tokyo, Japan). Inotodiol was purchased from ALB Technology Ltd. (Henderson, NV, USA). Diaion HP-20, formic acid, glacial acetic acid (99.7%), and perchloric acid (70%) were purchased from Samchun Chemical Co. (Seoul, Korea). Acetonitrile, *n*-butanol, and methanol were from JT Baker (Phillipsburg, NJ, USA). Cancer cell lines (HT-29, AGS, MCF-7, and PC3) and the macrophage cell line (RAW 264.7) were obtained from Korea Cell Line Bank (Seoul, Korea). Roswell Park Memorial Institute (RPMI) 1640, Dulbecco’s modified Eagle medium (DMEM), and phosphate buffered saline (PBS) were purchased from GIBCO Invitrogen (Grand Island, NY, USA). Fetal bovine serum (FBS) and penicillin/streptomycin were purchased from WelGENE Inc. (Daegu, Korea) and GE Healthcare Life Sciences (South Logan, UT, USA), respectively. DMSO and MTT were purchased from Sigma-Aldrich Chemical Co. (St. Louis, MO, USA).

### 3.2. Material

Inner and outer parts of *I. obliquus* collected in Tyumen, Russia were provided from DHF Co. (Seoul, Korea). These parts were ground to a fine powder and stored at 15 °C with vacuum packing until being used for extraction.

### 3.3. Extraction and Fractionation

The dried powder (20 g) was refluxed with 180 mL 80% methanol for 24 h in a water bath (Daihan Scientific Co., Seoul, Korea) at 80 °C. The extract was filtered twice through a Whatman No. 4 filter paper (Whatman International Ltd., Maidstone, England). A part of the filtrate was dried (designated as the methanol extract) and stored before being used for the anti-proliferative activity assay. The rest of the filtrate was fractionated by the method of Shin et al. (2001) with a slight modification [21]. Briefly, the solvent in the filtrate was evaporated under reduced pressure at 40 °C to be 100 mL, followed by adding 100 mL water to the concentrated filtrate. The solution (200 mL) was extracted twice with water-saturated *n*-butanol (200 mL) in a separatory funnel, followed by collecting the *n*-butanol layer. Meanwhile, a glass column (inner diameter, 5 cm; length, 25 cm) was filled with Diaion HP-20 dispersed in methanol (200 mL) and left for 24 h in order to eliminate bubbles and impurities. The column was washed with water and methanol (1 L each) just before loading the sample, followed by subjecting the collected *n*-butanol layer into the column. The column was washed with 50% methanol (500 mL) and sequentially eluted with absolute methanol (500 mL) to obtain the triterpenoid fraction. The triterpenoid fraction was lyophilized and stored at −20 °C until further analysis. The dried triterpenoid fractions from the inner and outer parts of *I. obliquus* were designated as the IPTF and OPTF, respectively.

### 3.4. Determination of Total Triterpenoids

Total triterpenoids were determined according to the colorimetric method [15]. The extract dissolved in methanol (10 mg/mL) was vigorously shaken and centrifuged at 5000× *g* for 20 min. The supernatant (100 µL) was mixed with 150 µL 5% (*w*/*v*) vanillin-glacial acetic acid and 500 µL 70% perchloric acid. The solution was heated for 45 min at 60 °C and cooled down to room temperature. Glacial acetic acid (2.25 mL) was added to the solution and absorbance was measured at 548 nm using a UV–vis spectrometer (Spectramax 190, Molecular Devices, Sunnyvale, CA, USA). Total triterpenoids were expressed as UAE/g extract on a dry basis.

### 3.5. Analysis of Triterpenoid Composition

The IPTF or OPTF dissolved in methanol (10 mg/mL) were agitated using a sonicator (5510E-DHT, Bransonic, Danbury, CT, USA). The solution was centrifuged at 8000× *g* for 20 min at 4 °C. The supernatant was filtered with a 0.2 µm nylon syringe filter (Whatman International Ltd., Maidstone, England) and stored at −20 °C until analyzed.

Triterpenoids were identified using an LTQ XL (Thermo Fisher Scientific, Waltham, MA, USA) connected to an Ultimate 3000 RS system via the ESI interface. Stationary phase was a U-VDSpher PUR C18-E (1.8 µm, 100 × 2.0 mm, VDS Optilab, Berlin, Germany) and mobile phases were water with 0.1% formic acid (solvent A) and acetonitrile with 0.1% formic acid (solvent B). Flow rate was 0.3 mL/min with a gradient as follows: 0 min, 90% B; 0–10 min, 100% B; 10–22 min, 100% B; 22–23 min, 90% B; and 23–30 min, 90% B. Injection volume was 5 μL. Mass parameters were set as follows: detection ion mode, positive ([M + H, Na]^+^); scan range, *m*/*z* 100–2000; capillary temperature, 300 °C; source voltage, 3.5 kV; sheath gas flow, 42 arbitrary unit; and software, X-Calibur 2.0 (Thermo Fisher Scientific, Waltham, MA, USA).

Triterpenoids in *I. obliquus* were quantified using an HPLC (Waters Alliance 2695, Waters, Milford, MA, USA) equipped with a ZORBOX Eclipse Plus C18 (5 µm, 4.6 × 250 mm, Agilent, CA, USA). Mobile phases were water (solvent A) and acetonitrile (solvent B). Flow rate was 1.0 mL/min with a gradient as follows: 0 min, 90% B; 0–10 min, 97% B; 10–30 min, 97% B; 30–30.1 min, 90% B; and reconditioning for 9.9 min. Injection volume was 20 µL. Detection wavelength was set at 206 nm. Column oven was maintained at 30 °C. Triterpenoids were quantified by comparing peaks of corresponding standards on the HPLC chromatogram.

### 3.6. Cell Culture

HT-29 colorectal adenocarcinoma cells, AGS human gastric adenocarcinoma cells, MCF-7 breast adenocarcinoma cells, and PC3 prostatic adenocarcinoma cells were used for the anti-proliferative activity assay. These cell lines were cultured in RPMI 1640 containing 10% FBS and 1% penicillin/streptomycin. RAW 264.7 murine macrophage cells were used as a normal cell line. The cells were cultured in DMEM containing 10% FBS and 1% penicillin/streptomycin. All the cell lines were incubated in 5% CO_2_ at 37 °C and the cells with passage numbers between 2 and 10 were used for the assay.

### 3.7. Anti-Proliferative Activity

The MTT assay was performed to determine the anti-proliferative activity of the IPTF and OPTF on cancer cell lines in a concentration range that did not affect normal cells (RAW 264.7 cells). To compare the anti-proliferative activities of the triterpenoid fractions with those of the methanol extracts which had a higher amount of phenolic compounds and a lower amount of triterpenoids, anti-proliferative activities of the methanol extracts from the inner and outer parts of *I. obliquus* were also evaluated. Briefly, 2 ×10^4^ cells/well were seeded in a 96-well plate and incubated for 24 h in 5% CO_2_ at 37 °C. The methanol extracts and triterpenoid fractions were dissolved with DMSO (never excess 0.1%) and diluted with non-serum medium. Various concentrations (100–300 µg/mL) of the samples were treated on the cells and incubated for 24 h. The control was treated with non-serum medium containing 0.1% DMSO. After the incubation, the medium was discarded and MTT solution (100 µL) was added to each well, followed by incubation for 4 h. The MTT solution was replaced by 100 µL DMSO to solubilize formazan, followed by incubation for 20 min at room temperature. Absorbance was measured at 540 nm. Cell viability was calculated as percentage viability: (absorbance of treated cells/absorbance of control cells) × 100.

### 3.8. Statistics

All experiments were performed in triplicate. The results were expressed as means ± standard deviations. Data were subjected to one-way analysis of variance (ANOVA) with Duncan’s new multiple range test (*p* < 0.05) and Student’s *t*-test (*p* < 0.05 and *p* < 0.01) using SPSS 23.0 software (SPSS Inc., Chicago, IL, USA).

## 4. Conclusions

Triterpenoids from the inner and outer parts of *I. obliquus* were identified and their anti-proliferative activity was evaluated. Betulin, betulinic acid, inotodiol, and trametenolic acid were identified in both of the inner and outer parts of *I. obliquus*. Inotodiol and trametenolic acid were major triterpenoids. The anti-proliferative activity of the triterpenoid fraction from the outer part of *I. obliquus* was higher than that of the inner part. This might be because the amount of triterpenoids was higher in the outer part. The results of the study suggest that the outer part of *I. obliquus* may be used as functional foods or drugs as well as the inner part.

## Figures and Tables

**Table 1:** Extraction yields and total triterpenoid contents in *Inonotus obliquus.*

Fraction	Yield (%, *w*/*w*)	Triterpenoid Content (mg UAE/g)	
	Dry Basis of *I. obliquus*	Dry Basis of Fraction	Dry Basis of *I. obliquus*
IPTF	0.51 ± 0.04	469.2 ± 10.2	2.51 ± 0.04
OPTF	0.78 ± 0.14 *	554.5 ± 13.9 *	4.32 ± 0.11 *

Values are expressed as means ± standard deviations (*n* = 3). * Significant difference (*p* < 0.05). IPTF: triterpenoid fraction from inner part of *I. obliquus*; OPTF: triterpenoid fraction from outer part of *I. obliquus*; and UAE, ursolic acid equivalent.

**Table 2:** Triterpenoids in triterpenoid fractions from *Inonotus obliquus.*

Peak No. ^a^	Compound	Fraction (mg/g Dry Basis of Fraction)	
		IPTF	OPTF
1	Betulinic acid	8.69 ± 1.71	13.6 ± 2.29 *
2	Betulin	8.24 ± 0.65	8.79 ± 0.40
3	Trametenolic acid ^b^	94.5 ± 9.15	106.3 ± 8.23
4	Inotodiol	153.9 ± 15.4	194.1 ± 11.5 *
5	Unknown ^b^	59.0 ± 4.04	47.1 ± 15.7

Values are expressed as means ± standard deviations (*n* = 3). * Significant difference (*p* < 0.05). ^a^ Peak numbers of triterpenoids in HPLC chromatogram in Figure 2. ^b^ Trametenolic acid and an unknown compound were calculated as betulinic acid equivalent. IPTF: triterpenoid fraction from inner part of *I. obliquus*; and OPTF: triterpenoid fraction from outer part of *I. obliquus*.
